# A Genome-Wide Association Study for Resistance to Tropical Theileriosis in Two Bovine Portuguese Autochthonous Breeds

**DOI:** 10.3390/pathogens13010071

**Published:** 2024-01-12

**Authors:** Diana Valente, Octávio Serra, Nuno Carolino, Jacinto Gomes, Ana Cláudia Coelho, Pedro Espadinha, José Pais, Inês Carolino

**Affiliations:** 1 Centro de Investigação Vasco da Gama, Escola Universitária Vasco da Gama, 3020-210 Coimbra, Portugal; nuno.carolino@iniav.pt (N.C.); ines.carolino@iniav.pt (I.C.); 2 Escola de Ciências Agrárias e Veterinárias, Universidade de Trás-os-Montes e Alto Douro, Quinta de Prados, 5000-801 Vila Real, Portugal; accoelho@utad.pt; 3 Centro de Ciência Animal e Veterinária, Universidade de Trás-os-Montes e Alto Douro, Quinta de Prados, 5000-801 Vila Real, Portugal; 4 Instituto Nacional de Investigação Agrária e Veterinária, I.P., Banco Português de Germoplasma Vegetal, Quinta de S. José, S. Pedro de Merelim, 4700-859 Braga, Portugal; octavio.serra@iniav.pt; 5 Instituto Nacional de Investigação Agrária e Veterinária, Polo de Inovação da Fonte Boa—Estação Zootécnica Nacional, 2005-424 Santarém, Portugal; 6 Centro de Investigação Interdisciplinar em Sanidade Animal, Faculdade de Medicina Veterinária, Universidade de Lisboa, 1300-477 Lisboa, Portugal; 7 Laboratório Associado para a Ciência Animal e Veterinária, Faculdade de Medicina Veterinária, Universidade de Lisboa, 1300-477 Lisboa, Portugal; 8 Escola Superior Agrária de Elvas, Instituto Politécnico de Portalegre, 7350-092 Elvas, Portugal; 9 Associação de Criadores de Bovinos da Raça Alentejana, Monforte Herdade da Coutada Real-Assumar, 7450-051 Assumar, Portugal; 10 Associação de Criadores de Bovinos Mertolengos, 7006-806 Évora, Portugal; pais@mertolenga.com; 11 Instituto Superior de Agronomia, Universidade de Lisboa, 1349-017 Lisboa, Portugal

**Keywords:** tick-borne disease, *Theileria annulata*, single-nucleotide polymorphisms (SNPs), marker-assisted selection, Mertolenga breed, Alentejana breed

## Abstract

The control of Tropical Theileriosis, a tick-borne disease with a strong impact on cattle breeding, can be facilitated using marker-assisted selection in breeding programs. Genome-wide association studies (GWAS) using high-density arrays are extremely important for the ongoing process of identifying genomic variants associated with resistance to *Theileria annulata* infection. In this work, single-nucleotide polymorphisms (SNPs) were analyzed in the Portuguese autochthonous cattle breeds Alentejana and Mertolenga. In total, 24 SNPs suggestive of significance (*p* ≤ 10^−4^) were identified for Alentejana cattle and 20 SNPs were identified for Mertolenga cattle. The genomic regions around these SNPs were further investigated for annotated genes and quantitative trait loci (QTLs) previously described by other authors. Regarding the Alentejana breed, the *MAP3K1*, *CMTM7*, *SSFA2*, and *ATG13* genes are located near suggestive SNPs and appear as candidate genes for resistance to Tropical Theileriosis, considering its action in the immune response and resistance to other diseases. On the other hand, in the Mertolenga breed, the *UOX* gene is also a candidate gene due to its apparent link to the pathogenesis of the disease. These results may represent a first step toward the possibility of including genetic markers for resistance to Tropical Theileriosis in current breed selection programs.

## 1. Introduction

Tropical Theileriosis is a tick-borne disease caused by a hemoprotozoan, *Theileria annulata*, which affects cattle production in Europe, Asia, and Africa [1]. This parasite can affect the host’s immune system and cause damage to red blood cells, leading to varying degrees of anemia [2]. This will result in production shortfalls and increased mortality and morbidity rates of parasitized animals. Thus, Tropical Theileriosis causes economic losses associated with reduced production capacity and the need to carry out control for diseases and ticks; for example, through the use of acaricides [3].

Theileriosis control strategies may include curing affected animals and prevention [4]. Curing affected animals can be difficult and involves the administration of substances such as imidocarb, erythromycin, oxytetracycline, quinozoline, and naphthoquinone derivatives (parvaquone and buparvaquone), whose efficacy is questionable [5,6,7]. In addition, buparvaquone, for example, can leave residues in animal products, such as meat and milk, implying long safety intervals, and its use is, therefore, limited in several countries, including European countries, where its use is not approved by the EMA (European Medicines Agency) [5,8]. In an attempt to control this disease, where it is difficult to achieve a cure, it is possible to resort to culling affected animals, although this results in significant economic losses for the farm [4]. The prevention of Tropical Theileriosis can be based on strategies that include the control of ticks (e.g., use of acaricides), the environment (e.g., biosecurity measures and avoiding movement of animals from non-endemic to endemic areas), and the animal (e.g., selection of resistant animals and use of vaccines) [4,5]. The use of acaricides in the control of ticks and tick-borne diseases is one of the main strategies used, but it is costly [4]. In addition, increasing acaricide resistance is also a concern in the implementation of this control strategy [4,7,9]. Acaricide resistance results from the selection of specific heritable traits in a tick population, resulting from exposure of the population to an acaricide. As a result, there will be an increase in the number of ticks that survive after the administration of the recommended dose of the same acaricide to which resistance already exists [9]. In turn, the use of vaccines, an animal-based control strategy for Tropical Theileriosis, also has some limitations. The only vaccines that appear to be effective are live attenuated vaccines, but more research is needed to fully understand their mechanism of action [5,7]. Subunit vaccines are difficult to obtain, considering the genetic diversity of these parasites [5]. Finally, the use of resistant animals could be another, more sustainable, strategy to minimize the impact of the Tropical Theileriosis [6,8].

The use of animals that are genetically tolerant/resistant to ticks and tick-borne diseases is thus considered the most natural control alternative [10]. Natural disease resistance refers to the inherent ability of an animal to resist diseases when exposed to pathogens without prior exposure or immunization [4]. Several authors have reported that cattle of autochthonous breeds from endemic regions are more resistant than those of exotic breeds [3,11,12,13]. For example, different responses to *T. annulata* infection have been identified in the Sahiwal breed (*B. indicus*) and the Holstein breed (*B. taurus*) [11,14]. 

An important prerequisite in breeding strategies for disease tolerance/resistance is the attempt to understand the genetic control mechanisms of diseases. Nowadays, it is known that tick loads in cattle have moderate to high levels of heritability, which can range from 0.40 to 0.54 [15,16]. In addition, some hemoparasitoses, such as those caused by *Anaplasma marginale* and *Ehrlichia ruminantium*, appear to have significant heritabilities, with values of 0.16 and 0.19, respectively [17]. Genome-wide association studies have been conducted for some vector-borne diseases [10]. In addition, single-nucleotide polymorphisms (SNPs) have been tested to identify genetic variants associated with complex traits. SNPs distributed throughout the genome can detect and map mutations underlying variation in target traits by a process called genome-wide association analysis (GWAS) [18]. GWAS allows for the identification of genetic markers, candidate genes, and QTLs for individual traits. The discovery of a quantitative trait locus (QTL) is an important step in identifying and understanding genetic variants associated with economically important phenotypes, and genome-wide association study (GWAS) has become a widely used approach for identifying QTLs and genome regions associated with phenotypes [19]. High-density arrays capable of genotyping thousands of SNPs are already available for cattle, allowing for increased genomic coverage and statistical power [20]. 

This study aims to identify SNP genetic markers that may be associated with resistance to Tropical Theileriosis in the cattle of the autochthonous Portuguese breeds Alentejana and Mertolenga and genes or genomic regions that are most likely involved in resistance to this disease. These data will be important information to include in breeding programs for these breeds, which are at risk of extinction and have conservation and improvement programs in place.

## 2. Materials and Methods

### 2.1. Sample Characterization

The blood samples used in this study were collected in the Alentejo region of Portugal, the country’s leading beef-producing region, with more than 65% of national production. This region is the place of origin of the Portuguese autochthonous breeds under study, namely the Alentejana and the Mertolenga breeds, and is also an endemic region for *T. annulata* [21]. The Alentejana and Mertolenga breeds belong to a group of 15 autochthonous Portuguese cattle breeds and are at risk of extinction [22]. The Alentejana cattle breed has around 22,000 breeding females registered in the herd book, spread over around 174 farms, although only around 8000 are purebred, and the rest are used for crossbreeding with males from exotic international breeds [23]. On the other hand, the Mertolenga cattle breed, which currently has 27,000 breeding females, is the largest of Portugal’s 15 autochthonous breeds. Similar to the Alentejana breed, only around 8000 females are purebred, the rest are used to crossbreed with males from exotic international breeds [24].

Initially, 843 blood samples were randomly collected from purebred Alentejana and Mertolenga cattle, which did not show any clinical signs of *T. annulata* infection. The collections were carried out between November 2018 and December 2019 in 420 Alentejana breed animals and 423 Mertolenga breed animals. These animals all belong to farms with extensive or semi-extensive production regimes, so these animals live outdoors and in a pasture. 

### 2.2. Sample Collection

Between 3 and 5 mL of blood were collected from the jugular vein of each animal and stored individually in tubes containing EDTA (ethylenediaminetetraacetic acid). This blood sampling was carried out by the technicians of the cattle associations of the autochthonous breeds under study. The tubes containing the blood sample were frozen at −20 °C before being sent to the laboratory. During transport and storage, the temperature conditions were maintained.

### 2.3. Sample Processing

Initially, these samples were analyzed at the Molecular Genetics Laboratory of the National Institute for Agricultural and Veterinary Research (INIAV). In the laboratory, 300 µL of each of the 843 blood samples were used to perform DNA extraction using a Cytogene^®^ Blood Kit (Lucknow, India, Cytogene), following the manufacturer’s instructions. Thereafter, DNA from each sample was subjected to amplification with the Polymerase Chain Reaction (PCR) of a fragment, with about 319 base pairs (bp), of a *T. annulata* merozoite-piroplasm surface antigen gene, *Tams 1* [25]. In all reactions, a negative sample of *T. annulata* (negative control) was used, without DNA, and a positive sample (positive control), property of the Parasitology Laboratory of the INIAV (Lisbon, Portugal). The amplified samples were analyzed by 1.5% agarose gel electrophoresis, and the gel was visualized with an ultraviolet (UV) transilluminator. Positive and negative PCR controls were introduced in each PCR, and a molecular weight marker (NZYDNA VI) was placed on each gel. Samples were classified as positive (samples infected with *T. annulata*, with the presence of the *Tams 1* gene) and negative (samples not infected with *T. annulata*, without the presence of the *Tams 1* gene). From the 843 blood samples analyzed, 96 samples from animals of the Alentejana breed and 96 samples from animals of the Mertolenga breed were selected. In the case of the animals of the Alentejana breed, all the samples positive for *T. annulata* (30 samples) were used, and the farms to which they belonged were identified. The remaining 66 negative samples were selected from the same farms as the previous ones. In the case of the Mertolenga breed, we used 48 samples from infected animals and 48 samples from non-infected animals (*n* = 96), selecting the latter based on the farm to which the infected animals belonged. The presence of the *Tams 1* gene (infected animals) or its absence (non-infected animals) were the phenotypic extremes considered in our study. Thus, infected animals, with the presence of the *Tams 1* gene in the blood sample, were considered susceptible to Tropical Theileriosis, while non-infected animals, without the presence of the *Tams 1* gene in the blood sample, were considered resistant to Tropical Theileriosis.

### 2.4. SNP Genotyping and GWAS Analysis

All 192 samples were genotyped in two plates of 96 samples in the Axiom™ Bovine Genotyping 100K Array, 1 plate for each breed, in a laboratory providing animal health and food safety services (Segalab, Portugal), following the best practices workflow from the manufacturer. The “BestandRecommended” SNPs were selected for the analysis of both breeds. PLINK v1.9 was used for quality control [26]. SNPs were filtered out whenever the minor allele frequency (--maf) was below 5% and missing data (--geno) was higher than 10%. Similarly, samples were filtered out when there was 10% or more missing data (--mind). To account for population structure, samples were filtered based on cryptic relatedness (--min 0.2), and their distribution was inferred by means of a principal component analysis (--pca var-wts). Whenever a principal component was responsible for a significative separation of samples into groups, which was not expected from sample records, it was used as a covariate for the association analysis. Genome-wide association analysis was performed in PLINK under the logistic model for disease traits (--logistic). Case/control phenotypes for *T. annulata* were included in the phenotype files (--pheno). The origin of each sample, namely its breeder or the district of provenance, was used as a covariate (--covar). The association was evaluated using quantile–quantile plots. The *p*-values for all SNPs were adjusted for the false discovery rate (FDR). Also, the threshold of significance was calculated following the Bonferroni correction.

### 2.5. Data Analysis

Data analysis was supported by querying the Bovine Mine and National Center for Biotechnology Information databases [27,28]. When analyzing the data using these databases, the aim was to identify the overlap of suggestive SNPs with possible genes and QTLs or to identify genes in their vicinity.

## 3. Results

### 3.1. Descriptive Statistics

In the sample initially used (*n* = 843), it was possible to find positivity to *T. annulata* of 7.10% (30/420) in Alentejana animals and 14.4% (61/423) in Mertolenga animals [21]. As mentioned above, we selected 96 Alentejana animals and 96 Mertolenga animals, whose characteristics are described in the table below (Table 1).

### 3.2. Genome-Wide Associations

To identify SNP genetic markers and QTLs for resistance to Tropical Theileriosis, we performed a GWAS using a genotyping approach. After implementing data quality control measures, out of the 100,000 SNPs on the array, 75,126 SNPs were used in the association test for the Alentejana breed and 81,357 SNPs were used for the Mertolenga breed, and the analysis was performed for each breed separately.

**Figure 1:**
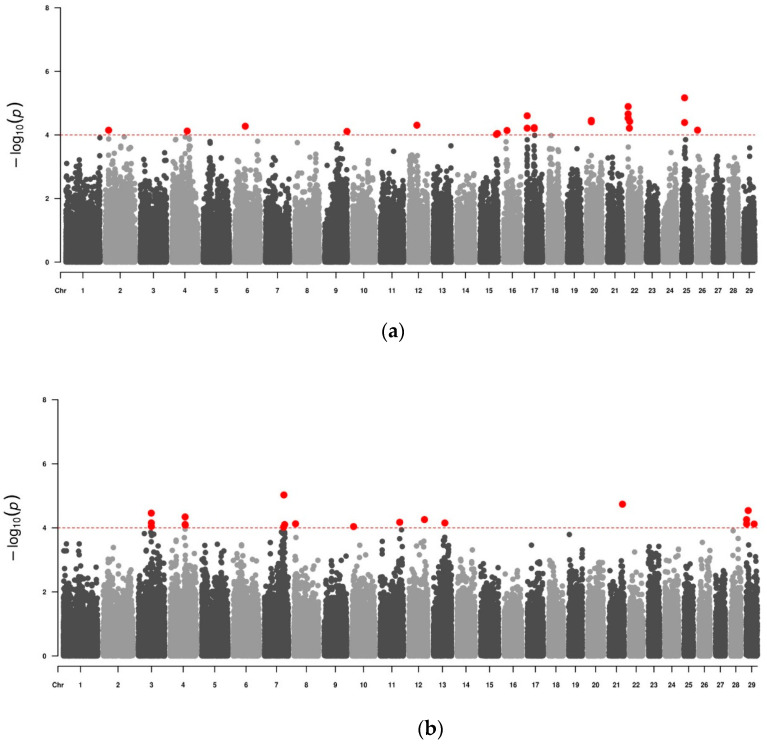
Manhattan plot of the association study for resistance to tropical theileriosis in two Portuguese autochthonous bovine breeds. The genome-wide significance threshold is indicated by the dashed line (*p* < 10^−4^). The red dots above the threshold line represent the SNPs considered as significant in this work. The position of the bovine chromosome is shown on the *x*-axis. The strength of association for a GWAS single-locus mixed model is shown on the *y*-axis. (**a**) Data from Alentejana breed animals. (**b**) Data from Mertolenga breed animals.

Considering an initial *p*-value ≤ 0.05, it was possible to find 7833 significant SNPs in the Alentejana breed and 7157 significant SNPs in the Mertolenga breed. All SNPs were classified as non-significant based on the adjusted *p*-values after the FDR. Furthermore, the Bonferroni correction also established a threshold of significance lower than the lowest *p*-value found. Despite this, based on other GWAS works available in the literature, it was decided that the suggestive value of significance to be used in this work would be *p*-value ≤ 10^−4^ (Figure 1) [29,30,31]. Thus, it was possible to find 24 significant SNPs for Alentejana cattle and 20 significant SNPs for Mertolenga cattle (Table 2). In this table, we can see that for both breeds, the protective allele of the identified SNPs appears in a higher percentage in the animals under study. Thus, we found that the protective allele appears more frequently in 75% of the SNPs studied in the Alentejana breed and 80% of the SNPs studied in the Mertolenga breed.

For the Alentejana breed, considering the 24 SNPs suggestive of genomic significance for resistance to Tropical Theileriosis, it was not possible to find any overlapping QTLs. On the other hand, in the case of the Mertolenga breed, considering the twenty SNPs suggestive of genomic significance, seven QTLs were found in the vicinity of the suggestive SNPs, distributed over four chromosomes (chromosomes 3, 7, 8, and 29) (Table 3).

In addition to the identification of QTLs based on SNPs suggestive of genomic significance, the genes where these SNPs overlapped with or the genes upstream and downstream of them, in the case of intergenic variants, were also considered. Thus, it was possible to identify different genes on chromosomes 2, 4, 6, 9, 12, 15, 16, 17, 20, 22, 25, and 26 for the Alentejana breed (Table 4) and on chromosomes 3, 4, 7, 8, 10, 11, 12, 13, 21, and 29 for the Mertolenga breed (Table 5).

## 4. Discussion

In this work, GWAS was performed using a high-density bovine SNP array that allowed the identification of 24 significant SNPs for Alentejana breed cattle and 20 significant SNPs for Mertolenga breed cattle (*p* ≤ 10^−4^). For these SNPs, in both breeds, we found that the protective allele is the most prevalent. In addition, only in the case of the Mertolenga breed was it possible to identify seven QTLs already described, associated with SNPs suggestive of resistance/susceptibility to Theileriosis. These QTLs are associated with traits such as average daily gain, muscle carnosine, creatine and creatinine content, body weight at birth, larger muscle area, and percentage of glycated kappa-casein in milk. These data are extremely important because the genetic selection of animals may be considered important health traits but cannot neglect the productive traits of these animals. Thus, the use of QTLs in genomic selection studies and breeding programs makes it possible to maximize genetic and economic gains [36]. Thus, these programs may allow the selection of animals with higher combined economic value for the next generation by combining productive and non-productive traits, such as resistance to Theileriosis [37]. In the case of the breeds under study, we think these data are even more valuable, as these are two autochthonous Portuguese breeds at risk of extinction. Thus, the selection of productive and healthy characteristics increases the interest of livestock producers in breeding these animals, which could contribute to an increase in the number of animals. On the other hand, selection for resistance to this disease contributes to animal health and welfare and to reducing the use of chemical substances as an approach to treating and controlling Tropical Theileriosis.

It was also possible to identify several annotated genes that overlap with the suggestive SNPs from GWAS, or in their vicinity. For the Alentejana breed, it was possible to find genes associated with the regulation of cell proliferation, differentiation, and survival, such as *EGFR* (*epidermal growth factor receptor*), cell growth, such as *DCHS2* (*dachsous cadherin-related 2*), and the formation of plasma cation channels, such as *TRPC3* (*transient receptor potential cation channel subfamily C member 3*) [38,39,40]. In addition to these functions at the cellular level, of the genes described, the *MAP3K1* (*mitogen-activated protein kinase kinase 1*) gene was identified, which acts in the MAPK pathway, a signal transduction pathway that modulates physiological and pathophysiological cellular responses. In this pathway, mitogen-activated protein kinases regulate important cellular processes such as proliferation, stress response, apoptosis, and immune defense, regulating the production of T helper 1 (Th1) and T helper 2 (Th2) lymphocyte responses. Currently, the ability of protozoan parasites such as *Trypanosoma cruzi*, *Trypanosoma congolense*, and *Leishmania* spp. to modulate the host immune response by intervening in the MAPK pathway to favor their replication and survival has been described [41,42]. Another gene recognized was *CMTM7* (*KLF-like MARVEL transmembrane domain-containing 7*), which belongs to the superfamily encoding chemokine-like factors. A lack of *CMTM7* has already been shown to cause a reduction in the innate B-cell population and lead to natural IgM and IL-10 deficiency [43]. IL 10 is one of the interleukins present in the highest concentration when cattle are infected with *T. annulata* and is essential in the development of the immune response against this agent [44]. In turn, the *SSFA2* (*sperm-specific antigen 2*) gene was identified, whose presence has already been reported to be associated with greater resistance to the development of clinical mastitis in cattle [45]. In addition to all these, the *ATG13* (*autophagy-related 13*) gene was also identified as being responsible for cellular autophagy, i.e., programmed cell death when cells are aged, degenerated, or non-functional. The action of this gene in the autophagy process in cells infected with the Bovine Viral Diarrhea Virus (BVDv) has already been described [46]. The BVDv is an intracellular pathogen at a certain stage of its cycle, like *T. annulata* [47].

Interestingly, two genes were identified in the group of Alentejana animals that seem to be associated with neurotransmitter concentration. It is known that the abnormal concentration of neurotransmitters is one of the factors that affect the health status, temperament, and welfare of animals, but the genetic basis of this abnormality is still unknown [48]. Despite this, there are already references that the *PCDH15* gene (*protocadherin-related 15*), which was identified in this work, may be associated with the regulation of this concentration. On the other hand, it was also possible to identify the *LOC107133268* gene (*protocadherin-16-like*), whose functions are not yet fully described in cattle, but the homolog, in humans, seems to be involved in the modulation of synaptic transmission and the generation of specific synaptic connections [49]. In addition, the genes *LOC107133203* (*olfactory receptor 1S1-like*) and *LOC104974344* (*olfactory receptor 10Q1*), which encode olfactory receptor proteins responsible for the recognition and transduction of olfactory signals, have also been identified and can trigger a neuronal response that triggers the perception of an odor [50].

In addition, we identified genes potentially associated with resistance to Theileriosis in Alentejana breed animals that appear to have significant productive importance, such as the *ATP12A* (*ATPase H/K transporting non-gastric alpha2 subunit*), *SHISA9* (*shisa family member 9*), and *FSTL5* (*follistatin-like 5*) genes. The *ATP12A* gene encodes the H+/K+ ATPase type 2 protein, a membrane protein involved in transmembrane cation transport, which is present in sperm and acts in the acrosome reaction at fertilization [51]. On the other hand, the *SHISA9* gene is associated with pre-weaning growth, while the *FSTL5* gene appears to be associated with muscle hypertrophy in cattle, inducing follistatin to increase insulin action at the skeletal muscle level [52,53,54,55]. Finally, the *BRINP3* (*BMP/retinoic acid inducible neural-specific 3*) gene was also identified, for which its association with meat quality and fertility in heifers is reported [56,57].

Regarding the genes identified, there is an overlap of two SNPs with the *SHISA9* and *EGFR* genes, which affect the animals’ productive capacity and cell regulation, respectively. In addition, one SNP overlaps with the *DCHS2*, *MAP3K1*, *CMTM7*, *PCDH15*, *SSFA2*, and *ATG13* genes, most of which act in cell regulation and protective cell response. All other genes described are near the SNPs identified.

In the case of the Mertolenga breed, it was also possible to identify genes with different functions. Thus, the UOX (Bos taurus urate oxidase) gene was identified, which encodes the urate oxidase enzyme and has the function of degrading uric acid, a hematological parameter that is increased in animals infected with *T. annulata* [58,59]. In addition, the *TMC2* (*transmembrane channel-like 2*) gene, which encodes proteins responsible for the formation of mechanosensitive ion channels at the tips of sensory cells in the inner ear, has been identified in mammals [60]. In humans, it is reported to be associated with hearing loss and epidermodysplasia verruciformis [61,62]. Cumulatively, the *ZBTB44* (*zinc finger and BTB domain-containing 44*) gene has been identified and appears to be associated with the macrophage-dependent immune response in *Mycobacterium avium* subsp. paratuberculosis infection in cattle [63].

Regarding genes associated with production, it was possible to identify the *EED* (*embryonic ectoderm development*) gene, whose association with the milk production capacity of cattle has already been reported [64]. In addition, the *LOC107131273* gene (*multidrug resistance-associated protein 4-like*) was identified, for which there is an indication of differential expression in cows pregnant with large fetuses, which leads to the development of dystocia, with significant impacts on production [65]. Furthermore, the *MTNR1B* (*melatonin receptor 1B*) gene has been identified, which is expressed in mammalian oocytes, and the *TRNAC-GCA* (*tRNA-Cys*) gene appears to be associated with sperm quality [66]. In addition to these, the *DRD1* (*dopamine receptor D1*) gene, which encodes the dopamine D1 receptor protein, has been identified. These receptors are prime candidates in the regulation of energy for the maintenance of homeostasis and are implicated in the regulation of feeding behavior in cattle [67]. Finally, the *MEF2C* (*myocyte enhancer factor 2C*) gene encoding a myocyte enhancer protein was identified and is important for skeletal, cardiac, and smooth muscle development [68,69].

For the Mertolenga breed, two SNPs were found to overlap with the *MEF2C* gene and one SNP with the *LOC170131273*, *UOX*, and *ZBTB44* genes. In addition, the *UOX* gene is also found to overlap with a QTL (associated with average daily gain). This gene is a strong candidate for resistance to Tropical Theileriosis due to the apparent link with the pathogenesis of the disease, warranting further investigation. In addition, the fact that it is related to a QTL associated with average daily gain could also be a good indicator, since one of the losses associated with infection by *T. annulata* is reduced productivity [1]. All other genes recorded are near the significant SNPs identified.

In beef cattle, it is known that it is of utmost importance to increase the ability to resist diseases, which is strongly associated with their immune performance. However, the productive traits of these animals cannot be disregarded, meaning that genes associated with the production of more muscle, better quality meat, or even milk in the case of suckler females are of utmost importance.

To the best of the authors’ knowledge, there has been no other published work analyzing GWAS data from the autochthonous Portuguese Alentejana and Mertolenga breeds. As such, this is a study that contributes to deepening genomic knowledge of Portuguese genetic resources. On the other hand, the authors are also unaware of any similar work aimed at associating resistance/tolerance to Tropical Theileriosis using GWAS. Despite this, it was found that some functions of genes associated with resistance to other parasitic diseases, such as Amoebiasis, Trypanosomosis, Toxoplasmosis, and Leishmaniasis, are also common to candidate genes for resistance to Theileriosis and were identified in this study. Thus, we highlighted the action on the cell membrane (*CMTM7*, *TMC2*, and *ATP12A* genes), ATP binding (*ATP12A* gene), and immune response (*MAP3K1*, *CMTM7*, *SSFA2*, *ATG13*, and *ZBTB44* genes) [70]. This work then made it possible to identify some SNPs suggestive of an association with resistance/tolerance to Tropical Theileriosis and the genes and QTLs that overlap or are in the vicinity. In the future, it is important to replicate the results presented here in another sample of animals to confirm the biological and/or metabolic action of the SNPs and QTLs indicated in beef cattle, and, in particular, in the autochthonous Portuguese breeds Alentejana and Mertolenga, as many of them have not yet been characterized, or the existing information is only described in other species. Furthermore, considering that resistance to Tropical Theileriosis is a polygenic trait, it would be interesting to develop a genomic selection study capable of providing information on the evolution of the genomic sites that control this trait and that could provide us with targets for the genetic selection of the most resistant animals [36].

## 5. Conclusions

In this work, 24 candidate SNPs for resistance to *T. annulata* infection were identified in the Portuguese Alentejana autochthonous breed and 20 candidate SNPs were identified in the Portuguese Mertolenga autochthonous breed. For both breeds, the protective allele of the identified SNPs appears at a higher percentage in the animals under study. Also, seven QTLs were found in the Mertolenga breed, of which one overlaps with the candidate gene *UOX*. This gene appears to be associated with the pathogenesis of Tropical Theileriosis. In the case of the Alentejana breed, the *MAP3K1*, *CMTM7*, *SSFA2*, and *ATG13* genes will be good candidate genes for resistance to Tropical Theileriosis due to their importance in regulating the immune response or their already described impact on resistance to other diseases. Thus, due to the importance that these genes seem to have in Tropical Theileriosis, further studies will be required, focusing on the SNPs identified in this work. In this way, this study is the first step in identifying markers that could be applied in breeding programs for both breeds under study.

## Figures and Tables

**Table 1:** Characteristics of the Alentejana and Mertolenga animals under study.

Parameter		Alentejana	Mertolenga
***T. annulata*** **Infection**	**Positive**	30 (31.25%)	48 (50.00%)
	**Negative**	66 (68.75%)	48 (50.00%)
**Age**	**Youngest animal**	8 months	1 month
	**Oldest animal**	14 years and 2 months	8 years and 5 months
**Sex**	**Male**	5 (5.20%)	3 (3.10%)
	**Female**	91 (94.8%)	93 (96.90%)
**Number of farms**		14	13

**Table 2:** SNPs suggestive of genome-wide significance, with *p*-values and the allele associated with resistance to Tropical Theileriosis (Chr—chromosome; A1—minor allele; BETA—regression coefficient).

Alentejana Breed							
Marker	Chr	Position/ Variant	*p*-Value	A1	BETA	Protective Allele	Reference Allele/Alternative Allele
**rs29016369**	25	11866965 Intergenic	6.80 × 10^−6^	T	2.045	C	C/T
**rs382748014**	22	1—175689 Intergenic	1.28 × 10^−5^	A	2.190	G	A/G
**rs136686350**	22	1079338 Intron	2.19 × 10^−5^	T	2.162	C	C/T
**rs42824138**	17	3176994 Intron	2.50 × 10^−5^	A	1.767	G	A/G
**rs42349624**	22	1012010 Intron	2.97 × 10^−5^	C	2.017	T	T/C
**rs110733319**	22	1130073 Intergenic	2.97 × 10^−5^	T	2.017	G	G/T
**rs41580257**	20	22411430 Intron	3.48 × 10^−5^	A	2.100	G	G/A
**rs109220983**	22	8175191 Intergenic	3.77 × 10^−5^	C	2.152	T	T/C
**rs110456301**	20	22270511 Intergenic	3.85 × 10^−5^	T	1.842	G	T/G
**rs136677596**	20	22278044 Upstream gene variant	3.85 × 10^−5^	T	1.842	C	T/C
**rs134209239**	25	11945164 Intergenic	4.08 × 10^−5^	C	1.751	T	C/T
**rs110136753**	12	36966751 Intergenic	4.94 × 10^−5^	T	1.621	C	T/C
**rs137404731**	6	49298093 Intergenic	5.33 × 10^−5^	A	−2.453	A	G/A
**rs135649048**	17	35855903 Intergenic	5.89 × 10^−5^	T	1.481	C	C/T
**rs110248505**	17	3089327 Intron	6.12 × 10^−5^	A	1.688	G	A/G
**rs378877063**	22	6853730 Intron	6.12 × 10^−5^	T	1.937	C	C/T
**rs133854848**	17	35658672 Intergenic	6.29 × 10^−5^	A	1.615	G	G/A
**rs42082138**	26	4547983 Intergenic	7.09 × 10^−5^	A	−1.996	A	G/A
**rs42238085**	2	14716248 Intron	7.11 × 10^−5^	A	−1.701	A	G/A
**rs134193812**	16	15787680 Intergenic	7.24 × 10^−5^	C	−1.530	C	C/T
**rs137244944**	4	70697022 Upstream gene variant	7.53 × 10^−5^	G	−1.827	G	G/A
**rs41657708**	9	103006480 Intergenic	7.74 × 10^−5^	T	−1.743	T	C/T
**rs136575474**	15	81387903 Intron	8.99 × 10^−5^	G	1.340	T	G/T
**rs41782203**	15	76586569 Intron	9.60 × 10^−5^	A	1.656	G	G/A
**Mertolenga Breed**							
**rs41625107**	7	89404622 Intergenic	9.40 × 10^−6^	G	1.584	A	A/G
**rs385061716**	21	62178639 Intergenic	1.82 × 10^−5^	T	1.776	C	C/T
**rs137077511**	29	9372082 Intergenic	2.88 × 10^−5^	T	1.638	G	T/G
**rs210844007**	3	59423141 Intron	3.46 × 10^−5^	G	1.580	A	A/G
**rs136003479**	4	68835630 Downstream gene variant	4.55 × 10^−5^	A	1.330	G	G/A
**rs42966583**	12	71327190 Intron	5.51 × 10^−5^	A	−1.378	A	A/C
**rs109210201**	29	1806321 Intergenic	5.53 × 10^−5^	C	1.784	A	C/A
**rs110183014**	11	89708117 Intergenic	6.69 × 10^−5^	A	−1.494	A	G/A
**rs43338206**	3	59696522 Intron	7.01 × 10^−5^	T	1.532	C	T/C
**rs110582951**	13	52653719 Intergenic	7.05 × 10^−5^	G	1.397	A	G/A
**rs41588323**	8	7368884 Intergenic	7.46 × 10^−5^	C	2.140	T	T/C
**rs42492357**	29	1982095 Intergenic	7.59 × 10^−5^	G	1.490	A	A/G
**rs41651830**	29	36432759 Upstream gene variant	7.59 × 10^−5^	C	1.490	T	T/C
**rs109072096**	4	68841016 Upstream and Downstream gene variant	7.84 × 10^−5^	A	1.246	G	G/A
**rs109619291**	7	92837313 Intergenic	7.91 × 10^−5^	T	1.301	C	C/T
AX-212327028 *	4	69412929 *	8.15 × 10^−5^	C	2.039	A	A/C
**rs43338222**	3	59690463 Missense variant and intron	9.01 × 10^−5^	T	1.508	C	C/T
**rs110062605**	10	6197570 Intergenic	9.14 × 10^−5^	A	1.340	C	C/A
**rs133541497**	7	88255064 Intron	9.41 × 10^−5^	A	−1.278	A	C/A
**rs137232658**	7	88256048 Intron	9.41 × 10^−5^	G	−1.278	G	A/G

* No rsID SNP or variant could be found; thus, the Affymetrix Axiom Bovine ID was presented.

**Table 3:** Quantitative trait loci already described that were found based on the SNPs with genomic significance considered for the Mertolenga breed (Chr—chromosome).

Marker	Chr	QTL ID	QTL Trait	References
**rs43338206**	3	22779	Average daily gain *B. taurus*	[32]
**rs41625107**	7	151659	Muscle carnosine content	[33]
**rs41625107**	7	151673	Muscle creatine content	[33]
**rs41625107**	7	164401	Body weight (birth)	[34]
**rs41625107**	7	164580	Longissimus muscle area	[34]
**rs41588323**	8	151760	Muscle creatinine content	[33]
**rs137077511**	29	116645	Milk glycosylated kappa-casein percentage	[35]

**Table 4:** Identification of genes using SNPs with significance for the Alentejana breed (*Bos taurus*) (Chr—chromosome).

Marker	*p*-Value	Chr	Position	Gene	Gene Location (bp) (start-end)
**rs29016369**	6.80 × 10^−6^	25	11866965	*SHISA9*—*shisa family member 9*	11633241-11647753
					11633245-12158399
**rs382748014**	1.28 × 10^−5^	22	1175689	*EGFR*—*epidermal growth factor receptor*	905960-1121554
				*RF00003*	1258027-1258168
**rs136686350**	2.19 × 10^−5^	22	1079338	*EGFR*—*epidermal growth factor receptor*	905960-1121554
**rs42824138**	2.50 × 10^−5^	17	3176994	*ENSBTAG00000024545*	3009649-3332773
				*DCHS2—dachsous cadherin-related 2*	3137604-3333575
**rs42349624**	2.97 × 10^−5^	22	1012010	*EGFR—epidermal growth factor receptor*	899974-1121540
					905960-1121554
**rs110733319**	2.97 × 10^−5^	22	1130073	*EGFR—epidermal growth factor receptor*	899974-1121540
					905960-1121554
				*RF00003*	1258027-1258168
**rs41580257**	3.48 × 10^−5^	20	22411430	*MAP3K1—mitogen-activated protein kinase kinase kinase 1*	22340163-22417428
**rs109220983**	3.77 × 10^−5^	22	8175191	*ENSBTAG00000051119*	8163685-8167126
				*RF00026*	8222734-8222838
**rs110456301**	3.85 × 10^−5^	20	22270511	*LOC112443032—uncharacterized*	22159612-22183599
				*MIER3—MIER family member 3*	22279228-22305654
**rs136677596**	3.85 × 10^−5^	20	22278044	*LOC112443032—uncharacterized*	22159612-22183599
				*MIER3—MIER family member 3*	22279228-22305654
**rs134209239**	4.08 × 10^−5^	25	11945164	*SHISA9—shisa family member 9*	11633245-12158399
**rs110136753**	4.94 × 10^−5^	12	36966751	*ATP12A—ATPase H/K transporting non-gastric alpha2 subunit*	36642635-36664187
				*ENSBTAG00000049928*	37397698-37398144
**rs137404731**	5.33 × 10^−5^	6	49298093	*RF00156*	49020641-49020774
				*RF00019*	49412387-49412498
**rs135649048**	5.89 × 10^−5^	17	35855903	*TRPC3—transient receptor potential cation channel subfamily C member 3*	35728366-35800211
				*FSTL5—follistatin-like 5*	36260758-37189201
**rs110248505**	6.12 × 10^−5^	17	3089327	*LOC107133268—protocadherin-16-like*	3009266-3011829
				*ENSBTAG00000024545*	3009649-3332773
**rs378877063**	6.12 × 10^−5^	22	6853730	*CMTM7—Bos taurus CKLF-like MARVEL transmembrane domain-containing 7*	6821672-6869727
					6821790-6869725
**rs133854848**	6.29 × 10^−5^	17	35658672	*LOC782754—mpv17-like protein 2*	35638835-35639137
				*TRPC3—transient receptor potential cation channel subfamily C member 3*	35728366-35800211
**rs42082138**	7.09 × 10^−5^	26	4547983	*PCDH15—protocadherin-related 15*	4509270-5569299
**rs42238085**	7.11 × 10^−5^	2	14716248	*SSFA2—sperm-specific antigen 2*	14654230-14750696
				*ITPRID2—Bos taurus ITPR interacting domain-containing 2*	14654230-14751034
**rs134193812**	7.24 × 10^−5^	16	15787680	*BRINP3—BMP/retinoic acid inducible neural specific 3*	15141936-15631140
				*RF00001*	16102665-16102785
**rs137244944**	7.53 × 10^−5^	4	70697022	*C4H7orf31—chromosome 4 C7orf31 homolog*	70687353-70727845
**rs41657708**	7.74 × 10^−5^	9	103006480	*LOC101907681—uncharacterized*	102848965-102870347
				*LOC112448098—uncharacterized*	103129637-103132019
**rs136575474**	8.99 × 10^−5^	15	81387903	*LOC107133203—olfactory receptor 1S1-like*	81383730-81385093
				*LOC104974344—olfactory receptor 10Q1*	81397109-81421347
**rs41782203**	9.60 × 10^−5^	15	76586569	*ATG13—Bos taurus autophagy-related 13*	76578054-76620037
					76578279-76620035

**Table 5:** Identification of genes using SNPs with significance for the Mertolenga breed (*Bos taurus*) (Chr—chromosome).

Marker	*p*-Value	Chr	Position	Gene	Gene Location (bp) (start-end)
**rs41625107**	9.40 × 10^−6^	7	89404622	*MIR3660*—*microRNA 3660*	89558103-89558182
				*ENSBTAG00000048981*	88840369-88848347
**rs385061716**	1.82 × 10^−5^	21	62178639	*RF00003—RNA, U1 small nuclear 85, pseudogene*	62091713-62091865
				*LOC112443166—uncharacterized*	62865898-62974123
**rs137077511**	2.88 × 10^−5^	29	9372082	*EED—embryonic ectoderm development*	9265439-9296624
				*LOC112444890—uncharacterized*	9426391-9430563
**rs210844007**	3.46 × 10^−5^	3	59423141	*SSX2IP—Bos taurus SSX family member 2 interacting protein*	59398296-59456577
**rs136003479**	4.55 × 10^−5^	4	68835630	*RF02043*	68836226-68836391
**rs42966583**	5.51 × 10^−5^	12	71327190	*ENSBTAG00000026070*	71263913-71411435
				*LOC107131273—multidrug resistance-associated protein 4-like*	71265415-71400436
**rs109210201**	5.53 × 10^−5^	29	1806321	*SLC36A4—solute carrier family 36 member 4*	1701573-1743386
				*MTNR1B—melatonin receptor 1B*	1901714-1916511
**rs110183014**	6.69 × 10^−5^	11	89708117	*RF00017—RNA, 7SL, cytoplasmic 825, pseudogene*	89685923-89686207
				*ENSBTAG00000052434*	89823864-89861691
**rs43338206**	7.01 × 10^−5^	3	59696522	*UOX—Bos taurus urate oxidase*	59636716-59736408
				*DNASE2B—deoxyribonuclease 2 beta*	59681735-59700078
				*RPF1—ribosome production factor 1 homolog*	59600697-59617167
**rs110582951**	7.05 × 10^−5^	13	52653719	*TMC2—transmembrane channel-like 2*	52539174-52640959
				*SNRPB—small nuclear ribonucleoprotein polypeptides B and B1*	52666459-52675562
**rs41588323**	7.46 × 10^−5^	8	7368884	*ENSBTAG00000039873*	7325501-7331864
				*TRNAC-GCA—tRNA-Cys*	7430900-7430971
**rs42492357**	7.59 × 10^−5^	29	1982095	*MTNR1B—melatonin receptor 1B*	1901714-1916511
				*FAT3—FAT atypical cadherin 3*	1991657-2638923
**rs41651830**	7.59 × 10^−5^	29	36432759	*ZBTB44—zinc finger and BTB domain-containing 44*	36401292-36460165
					36408247-36429241
**rs109072096**	7.84 × 10^−5^	4	68841016	*RF02041*	68840319-68840692
				*RF02040*	68842022-68842077
**rs109619291**	7.91 × 10^−5^	7	92837313	*ENSBTAG00000054282*	92782983-92822683
				*LOC100848699—uncharacterized*	92887690-92904398
AX-212327028 *	8.15 × 10^−5^	4	69412929	*RF00100*	69383582-69383863
				*LOC112446335—uncharacterized*	69487699-69491861
**rs43338222**	9.01 × 10^−5^	3	59690463	*DNASE2B—deoxyribonuclease 2 beta*	59681735-59700078
					59681727-59699694
**rs110062605**	9.14 × 10^−5^	10	6197570	*DRD1—Bos taurus dopamine receptor D1*	5715882-5718113
				*LOC112448549—uncharacterized*	6357822-6363522
**rs133541497**	9.41 × 10^−5^	7	88255064	*MEF2C—myocyte enhancer factor 2C*	88250023-88407702
**rs137232658**	9.41 × 10^−5^	7	88256048	*MEF2C—myocyte enhancer factor 2C*	88250023-88407702

* No rsID SNP could be found; thus, the Affymetrix Axiom Bovine ID was presented.

## Data Availability

The datasets presented in this article are not readily available because the data are part of an ongoing study.
